# Cost-analysis and effectiveness of one-stage laparoscopic versus two-stage endolaparoscopic management of cholecystocholedocholithiasis: a retrospective cohort study

**DOI:** 10.1186/s12893-017-0274-2

**Published:** 2017-07-06

**Authors:** Anne Mattila, Johanna Mrena, Ilmo Kellokumpu

**Affiliations:** 1 Department of Surgery, Central Hospital of Central Finland, Keskussairaalantie 19, 40640 Jyväskylä, Finland; 2 Department of Surgery, Central Hospital of Central Finland, 40640 Jyväskylä, Finland

**Keywords:** Laparoscopy, Choledocholithiasis, Cost-analysis

## Abstract

**Background:**

One–stage laparoscopic common bile duct (CBD) stone clearance and laparoscopic cholecystectomy (LCBDE+LC) for cholecystocholedocholithiasis ( CCL) can be performed with similar short and long-term outcomes than two-stage endoscopic retrograde cholangiography followed by subsequent LC (ERCP+LC). This study examined retrospectively the outcome and hospital costs of one-stage versus two-stage treatment of CBD stones.

**Methods:**

From January 1999 and December 2014, 217 consecutive, elective patients underwent one-stage (LCBDE + LC ) or two-stage (ERCP + subsequent LC ) treatment for CBD stones. The data from the one-stage management was collected prospectively, and from the two-stage management retrospectively. The main measure of outcome was hospital costs, with the success of one-stage versus two-stage management, postoperative morbidity and postoperative stay as secondary outcome measures.

**Results:**

One-stage laparoscopic transcystic management was the least costly option compared to laparoscopic one-stage transductal approach (TC 5455€ versus TD 9364, *p* < 0.001) or two-stage management (6913 €, *p *= 0.02). Overall success rate of primary intervention (including conversions to open surgery) for CBD stone clearance was 96.9%, 97.0% and 98.3% after transcystic one-stage, transductal one-stage and two-stage approach, *p *= 0.79. Postoperative morbidity was 15.5% versus 7.5%,* p* = 0.64, and postoperative hospital stay median 2 days (IQR 2–5) versus 4.5 days ( IQR 3–7), *p* < 0.001 in the one-stage and two-stage management groups.

**Conclusions:**

Our study shows that laparoscopic one-stage transcystic management of CCL results in high rate of CBD clearance, fewer procedures per patient, shorter hospital and lower costs than the two-stage management. Therefore the one-stage transcystic management seems to be an attractive strategy for the treatment of CCL depending on local resources and surgical expertise .

## Background

CBD stones are commonly managed with pre-, intra or post-operative ERCP although laparoscopic common bile duct exploration (LCBDE) has gained wide acceptance over the last 20 years [1].

Current evidence demonstrates similar CBD stone clearance rate for LCBDE (75% -100%) and ERCP (62% -96%) [2–10].The advantages of LCBDE + LC include a reduced number of procedures and shorter hospital stay [3, 9, 10].

Two- stage treatment is currently the most commonly used strategy for of CCL. Costs of one-stage versus two-stage treatment of CBD stones, however, are scantily reported in the literature. Two randomized studies have reported in-hospital costs in favour of one –stage method [3, 10]. Non-randomized studies using propensity score or cost analysis have also shown lower total in-hospital costs for one-stage than for two-stage method [11–15].

In order to rationalize the treatment of CCL the aim of this study was to compare the success and costs of one-stage versus traditional two-stage management for CCL. Based on previous studies (3,10) our hypothesis was that the laparoscopic one-stage treatment is more cost-efficient, and is associated with an improved outcome and shorter hospital stay.

## Methods

Finland offers its residents government-subsidised public-sector specialised healthcare. Central Hospital of Central Finland hospital is a university-affiliated secondary referral center, and the only hospital offering surgical and advanced endoscopic service in the catchment area of 276,000 inhabitants.

**Fig. 1:**
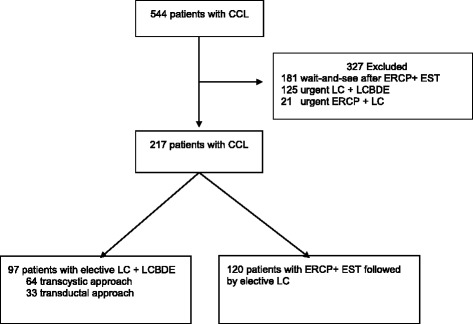
Flow chart of patients. CCL = cholecystocholedocholithiasis

From January 1999 to December 2014, alltogether 217 consecutive, elective patients with gallbladder stones and concomitant CBD stones were treated in our hospital. The one-stage group consisted of 97 consecutive patients who underwent LCBD exploration and concomitant LC in elective setting, with preoperative or intraoperative confirmation of choledocholithiasis. The two-stage group consisted of 120 consecutive patients with CCL who underwent preoperative ERCP + ES followed by elective LC. The flow chart of patients is presented in Fig. 1. Excluded from the study were patients who were scheduled for emergency LC due to acute cholecystitis, patients considered unfit for surgery or those few who refused cholecystectomy after ERCP and EST, and patients needing urgent ERCP for acute cholangitis.

CBD stones were diagnosed on the basis of clinical, laboratory, ultrasonographic, intraoperative cholangiography (IOC), choledochoscopy and since 2002 preoperative magnetic resonance cholangiopancreaticography (MRCP) findings. Before the MRCP era ERCP was occasionally used to diagnose and treat CBD stones.

The main measure of outcome was hospital costs per patient including readmissions. The cost analysis was undertaken from the perspective of healthcare providers view. The secondary measures of outcome were the success of CBD clearance, 30-day morbidity, mortality and length of postoperative hospital stay. Preoperative and short term outcome data of the one-stage group were collected prospectively, and similar data of the two-stage group retrospectively from hospital records. Patients from both study groups were evenly distributed during the observation period. Long-term outcome was investigated retrospectively using a mailed, self-completed questionnaire about jaundice, diagnosis and treatment of recurrent CBD stones as well as the date of diagnosis of recurrent stones. The causes of death were obtained from the National Cause of Death Registry. The study was approved by Ethics Committee of the Central Hospital of Central Finland. Informed consent was obtained from the prospective part of the study population. The need for informed consent from the retrospective patient cohort was waived. The aims and content of this study are in accordance with the Helsinki Declaration.

### **Surgical and endoscopic technique**

The decision to do one-stage or two-stage procedure was made according to surgeons experience and preference. Technique of LCBD exploration has been described in previous publications [16, 17] . In the one-stage group the primary aim was to do transcystic CBD stone clearance, when feasible. Transductal approach was chosen for large stones (>7–8 mm), multiple stones, if the CBD stones were situated above the cystic duct junction, if the cystic junction was posterior, or if the TC approach failed. Conversion to open surgery was made, if the laparoscopic one-stage CBD stone clearance failed, to avoid postoperative ERCPs, which also have a known morbidity and treatment failure rate [18]. LCBD explorations and LC were performed by senior surgeons or residents under senior guidance. All ERCP procedures were performed by senior surgeons familiar with the procedure. Patients underwent EST and clearance of CBD with balloon or Dormia basket. Laser lithotripsy was not available. In the case of residual CBD stones after primary ERCP clearance, a plastic stent was introduced and a new ERCP was scheduled.

### **Cost analysis**

Costs were calculated according to the year 2014 prices (€). Pre-existing data on some major resources and their allocated costs in 2014 were obtained from the hospital administration (Table 1). Costs of operative room resources (basic costs, anesthesia and nurses, surgical team, instrument use) and recovery room services were calculated according to the time spent in the operating and recovery rooms, duration of surgery, and the level of training required. The costs of disposable instruments including Dormia baskets, sphincterotomes, cannulas, extraction balloons, guidewires, stents, contrast agents, cholangiography catheters, trocars, drains, hemostatic agents, hemostatic sealing devices, and hemostatic clips for LCBD exploration in LC and ERCP were calculated according to the use. Excluded were costs of preoperative waiting time for the operation or ERCP, capital costs of reusable instruments, standard laparoscopic equipment, duodenoscopes, administration and societal costs. The correction coefficient of 0.82 for ERCP procedure price was based on the use of intravenous sedation without the presence of an anesthesiologist, permitting the ERCP time to be less costly than LC despite the same qualification of the attending surgeon.

**Table 1:** Major resources and their allocated costs

	Units costs (€)	
1. Operating rooms costs		
Basic costs (cleaning,electricity,sterilization,etc.)	197.72	
Total operating room time for LC (1 anesthesiologist +3 nurses)	8.5	per min
Total operating room time for ERCP (3 nurses without anesthesiologist)	0.82 × 8.5	per min
Operating time for specialist surgeon (LC)	0.6045	per min
Operating time for resident surgeon (LC)	0.403	per min
Recovery room time (LC)	0.50	per min
ERCP time for specialist surgeon	0.6045	per min
Recovery room time (ERCP)	0.50	per min
Equipment for LC and ERCP^a^		
2. Postprocedural costs		
Surgical ward after LC and ERCP^b^	602.00	per day
Intensive care unit^b^	1973.00	per day
T-tube cholangiography	234.00	
CT	135.00	
MRCP	258.00	
US	102.00	
Reoperation price^c^		
ReERCP price^c^		
Histology analysis (gallbladder)	350	
3. Readmission		
Outpatient physician consultation	176	
Readmission to surgical ward	602.00	
T-tube cholangiography	234	
CT	135.00	
MRCP	258.00	
US	102.00	
Reoperation price^c^		
ReERCP price^c^		

*ERCP* Endoscopic retrograde cholangiopancreatography, *MRCP* Magnetic-resonance cholangiopancreatography, *CT* Computed tomography, *US* Ultrasound

^a^Disposable instruments

^b^Personnel and overhead costs of surgical ward/ intensive care unit included

^c^Calculated separately depending on total operating room costs (paragraph 1)

### Statistical analysis

The data are presented as means with standard deviations (SD) or as medians with interquartile range (IQR) or as counts with percentages. Statistical comparison between the study groups was made by independent T-test, Mann-Whitney U test, Chi-Square test, the analysis of variance (ANOVA) or Kruskal-Wallis test, when appropriate. As the data for costs were highly skewed, bias corrected and accelerated bootstrap estimation was used to derive 95% confidence intervals and differences between the means were tested by bootstrap- type ANOVA, and post hoc testing of several univariate comparisons were made with Hochberg’s adjustment at significance level 0.05. The 95% confidence intervals (95%CI) are given for the most important outcomes. Statistical significance was defined as a *p* value <0.05. Statisical analyses were performed using SPSS statistical software (version 24.0 for Windows, SPSS Inc., Chicago IL, United States).

## Results

The flow chart of the patients is shown in Fig. 1. Alltogether 97 patients underwent one-stage and 120 patients two-stage treatment for CCL in the elective setting. Baseline characteristics are shown in Table 2. Patients in the two-stage group were slightly older with male predominance. Elderly patients ≥75 years and ASA –scores were evenly distributed in the study groups.

**Table 2:** Baseline characteristics

	One stage *N* = 97	Two stage *N* = 120	*P* value
Age, mean (SD) years	59.1 (19.0)	64.8 (15.3)	0.016
Age > 75, %	26 (26.8)	39 (32.5)	0.474
Male sex, n (%)	19 (19.6)	56 (46.7)	<0.001
BMI (kg/m^2^), mean (SD)	26.3 (3.9)	27.3 (5.4)	0.131
ASA, n (%)			
I -II	71 (73.2)	90 (75.0)	0.763

### Cost analysis

Overall, mean hospital costs per patient in the one-stage group were similar to the two-stage treatment group (6785 € versus 6913 €, *p* = 0.806) (Table 3). Additionally, when comparing patients with uncomplicated postprocedural course, the mean total costs were significantly lower in the one-stage group [5487 € (95% CI: 5164 € to 5809 €)] than in the two-stage group [6487 € (95% CI: 6019 € to 6956 €)], *p* < 0.001.

**Table 3:** Mean differences (95% CI) in hospital costs between one-stage and two-stage groups

Costs	One-stage *N* = 97		Two-stage *N* = 120	*P*-value (Multiple comparison)*
	Transcystic (TC) *N* = 46 Mean (95% CI)	Transductal (TD) *N* = 33 Mean (95% CI)	ERCP + LC *N* = 120 Mean (95% CI)	
Operation room, €	2806 (2680 to 2931)	3191 (3006 to 3377)	3025 (2898 to 3151)	TC/ TD 0.013 TC/ Two 0.071 TD/ Two 0.438
Postoperative, €	2572 (2200 to 2944)	5835 (3580 to 8089)	3825 (3309 to 4342)	TC/ TD <0.001 TC/ Two 0.049 TD/ Two 0.08
Readmission, €	77 (38 to 192)	339 (6 to 672)	63 (4 to 121)	TC/ TD 0.048 TC/ Two 0.018 TD/ Two 0.997
Total costs, €	5455 (4971 to 5938)	9364 (7048 to 11,681)	6913 (6340 to 7486)	TC/ TD <0.001 TC/ Two 0.029 TD/ Two 0.02

*CI* Confidence interval, *TC* Transcystic, *TD* Transductal

*Bias- corrected and accelerated bootstrap estimation was used to derive 95% confidence intervals

One-stage laparoscopic management using transcystic approach was the least costly option compared to laparoscopic one-stage transductal approach (TC 5455 € versus TD 9364 €, *p* < 0.001) or two-stage management (6913 €, *p* = 0.02) (Table 3). The per-patient operation room costs, postoperative expenses and readmission costs were lower in the one-stage transcystic group compared to one-stage transductal group. However, operation room and postoperative costs were similar, and readmission cost lower in two-stage treatment compared to one-stage laparoscopic transcystic treatment. Costs of disposable equipment were significantly higher in the two-stage group (739 €) compared to the one-stage group (526 €) due to a higher price of ERCP disposables, *p* < 0.001.

### Effectiveness of one-stage versus two-stage management

Short term outcome is shown in Table 4. Overall success rate of primary intervention (including conversions to open surgery) for CBD stone clearance was 96.9%, 97.0% and 98.3% after one-stage transcystic, one-stage transductal and two-stage approaches, *p* = 0.79. Conversion rate to open surgery was similar in the two study groups: one-stage group 8.2% (inflammation or insufficient visualization of the cystohepatic triangle 3, impacted stone in the ampulla 3, suspicion of CBD perforation 1 and residual CBD stones 1), two-stage group 13.3% (adhesions 14, intra-operative bleeding 1, and impacted stone 1). T-tube was inserted in 22 of the 33 patients (66.7%) who underwent transcholedochal stone clearance. The median total operative time was significantly shorter in the two-stage group compared to the one-stage group, *p* < 0.001. The 30-day morbidity was similar in the two study groups, with no difference in severe (Dindo-Clavien IIIb-V) complications. One patient died in the one-stage group due to Clostridium perfringens-sepsis. Postoperative hospital stay was median 2 days (IQR 2–5) versus 4.5 days (IQR 3–7), *p* < 0.001 in the one-stage and two-stage management groups. Reoperation rates were 4.1% in the one-stage group (intra-abdominal sepsis 2, bile leak from choledochotomy site 1 and duodenotomy leak after removal of impacted ampullary CBD stone 1), and 0.8% in the two-stage group (postoperative hemorrhage 1). The 30-day readmission rate was significantly higher in the one-stage group (19.6%) than in the two-stage group (5.8%), *p* = 0.002, due to more frequent ambulatory T-tube removal,and postoperative ERCPs (stent removal, suspicion of residual CBD stones and cystic stump leakage). In the two-stage group reasons for readmissions were ambulatory T-tube removal, postoperative wound infection, ERP for residual CBD stone and intractable postoperative pain (Table 4).

**Table 4:** Short-term (30-day) surgical outcome after one-stage and two-stage management

	One-stage *N* = 97		One-stage *N* = 97	Two-stage *N* = 120	*p*- value total one-stage vs two-stage
	TC approach *N* = 64	TD approach *N* = 33	Total		
Successful CBD stone clearance, n (%)					
LC + LCBDE /Index ERCP ^a^	59 (92.2)	27(81.8)	86 (88.7)	102 (85.0)	0.66
After conversion to open surgery	3(4.7)	5 (15.2)	8 (8.2)	16 (13.3)	0.36
After ERCP for residual stones	2 (3.1)	1 (3.0)	3 (3.1)	2 (1.7)	0.15
Total operative time, median (IQR) min ^b^	140 (69.3)	165 (52.5)	150 (61.0)	112 (64.0)	<0.001
30-d mortality, n (%) ^c^	0 (0)	1 (3.0)	1 (1.0)	0 (0)	0.27
30-d overall morbidity, n(%) ^d^	6 (9.4)	9 (27.2)	15 (15.5)	9 (7.5)	0.64
Surgical morbidity, n (%)	5(7.8)	4 (12.1)	9 (9.3)	5 (4.2)	0.13
Bile leak	2	3	5	0	
Postoperative bleeding	1	0	1	2	
Wound infection	1	0	1	1	
Intra-abdominal abscess	0	1	1	0	
Post-ERCP pancreatitis	1	0	1	0	
Bleeding after ERCP	0	0	0	2	
General morbidity, n (%)	1 (1.6)	5 (15.2)	6 (6.2)	5 (4.2)	0.50
Renal insufficiency	0	1	1	0	
Fever, unknown origin	0	0	0	1	
Vocal cord injury from intubation	0	1	1	0	
Myocardial infarction	0	0	0	1	
Heart insufficiency	1	0	1	1	
Pneumonia	0	3	3	2	
Dindo-Clavien gr IIIb-V, n (%)	2 (3.1)	3 (9.1)	5 (5.2)	2 (1.7)	0.25
Postoperative stay, median (IQR) days ^b^	2 (1–3)	5 (2–8)	2 (2–5)	4.5 (3–7)	<0.001
Reoperation, n (%)	2 (3.1)	2 (6.1)	4 (4.1)	1 (0.8)	0.11
Readmission, n(%)	3 (4.7)	16 (48.5)	19 (19.6)	7 (5.8)	0.002

*TC* transcystic, *TD* transductal

^a^Two-stage group

^b^Two-stage: ERCP and LC time

^c^Clostridium perfringens sepsis

^d^Figures in the columns are not additive because some patients had more than one complication

## Discussion

From a health economics point of view, two randomized trials from USA and India have demonstrated lower costs in the one-stage than in the two-stage management of CCL [3, 10] . This is in line to our study where transcystic approach resulted in lowest total costs. In the randomized trials [3, 10] patients had a good health status and younger age (median < 50 years) whereas in our study some 30% of the patients were older than 75 years and 25% of the patients had severe health problems (ASA III-IV), thus potentially increasing postoperative morbidity. It is well known that the clinical outcome and costs of surgery are dependant on surgeon’s experience and the quality of treatment. Some evidence of reduced hospital costs of one-stage treatment compared to two-stage treatment has also been reported in patients having uneventful post-procedural recovery [19]. When patients with postoperative complications were excluded in our study, the mean total difference was −1000 € in favor of the one-stage management.

Previous randomized trials and meta-analyses have demonstrated the safety and efficacy of one-stage management for CCL with a success rate of 75% to 96.8%, and with an associated postoperative morbidity of 3.6% to 43.2% [2–8, 10, 20]. Overall success of two –stage management has been 61.7% to 94.6%, with an associated postoperative morbidity of 5.1% to 29.8% [2–4, 7, 8, 10]. Our overall success rate for CBD stone removal and postoperative morbidity after one-stage and two-stage management are in accordance with these results. This was achieved with apparently similar surgical and ERCP-related morbidity.

Three of the 4 randomized trials reported longer total operative times in the two-stage management group [3, 7, 9, 10] in contrast to our study showing that one-stage management resulted in significantly longer operative time than the two-stage management. Conversion to transcholedochal approach after failed attempt of transcystic clearance increased the operative time in our study. Despite shorter total operative time in the two-stage management group, the operating room costs nested mainly from personnel expenditure of two separate procedures and disposable equipment used in ERCP.

Several studies have reported a significant reduction of hospital stay in patients receiving one-stage management compared with two-stage management of CBD stones [2–4, 7, 9, 10]. In accordance with these studies, the median postoperative hospital stay in our series was 2.5 days shorter in the one-stage group compared to the two-stage group. Postoperative hospital expenses originated mainly from basic surgical ward care accounting some 50% of the total costs of CBD stone management in both treatment groups. With this in mind, future efforts to improve hospital logistics and quality-of care are important to obtain shorter transit time and more profitable results. Accomplishing CBD stone treatment during single hospital visit should be a goal worth considering. Intraoperative ERCP (IOES) performed with rendez-vouz and assisted insertion of transcystic guidewire is considered as an improvement over standard ERCP techniques with lower rate of post-ERCP pancreatitis [21] So far, in randomised studies of one-stage management of CCL, only traditional IOES + LC versus LCBDE + LC have been compared with controversial results [22–24] A limitation of IOES is the requirement of simultaneous endoscopy team performing ERCP during laparoscopy in the operating theatre.

The proportion of readmission costs within total expenses were higher in the transductal group mainly due to ambulatory T- removal. However, the use of T-tube was dramatically reduced after reports on the safety of choledochotomy closure without T-tube [25].

Limitations and possible biases in this study are the lack of randomization which may have caused some selection bias, and the small number of patients making the detection of small differences between the study groups unreliable. The study design was retrospective and therefore cost-analysis instead of cost-benefit analysis was undertaken. Capital costs of laparoscopic equipment were excluded because laparoscopic equipment is nowadays considered standard operating room equipment used in many different operations. Costs in the Finnish healthcare are not applicable to every country, since the pricing of goods and services vary between healthcare systems. However, the share-out of the one-stage and two-stage management costs reflects the relative distribution of expenses between the the one-stage and two-stage management. Also the success rate of laparoscopic CBD stone clearance and hospital stay are in line with previous studies, suggesting that the quality of surgery has been as good as elsewhere.

## Conclusion

Our study shows that laparoscopic one-stage transcystic management of CCL results in high rate of CBD clearance, fewer procedures per patient, shorter hospital and lower costs than the two-stage management. Therefore the one-stage transcystic management seems to be an attractive strategy for the treatment of CCL depending on local resources and surgical expertise.

## Abbreviations

CBD: Common bile duct
CCL: Cholecystocholedocholithiasis
CI: Confidence interval
ERCP: Endoscopic retrograde cholangiopancreatography
IOES: Intraoperative endoscopic sphincterotomy
LC: Laparoscopic cholecystectomy
LCBDE: Laparoscopic common bile duct exploration
OR: Operating room
TC: Transcystic
TD: Transductal
